# Non‐selective cationic channels in chemical and physical stress

**DOI:** 10.1113/JP270937

**Published:** 2016-08-01

**Authors:** David J. Beech

**Affiliations:** ^1^ School of Medicine, University of LeedsLIGHT BuildingClarendon WayLeedsLS2 9JTUK

The symposium entitled “Non‐selective cationic channels in chemical and physical stress?”, which took place at Physiology 2015 in Cardiff, UK, 6–8 July 2015, lasted two hours and comprised five talks by invited speakers from France, Spain, the UK and USA who presented engagingly and informatively on concepts and new findings for seven proteins which assemble into what are thought to be seven distinct broadly expressed non‐selective cationic channels: polycystin‐2 channels, Piezo1 and Piezo2 channels, TRPA1 channels, TPC1 and TPC2 channels, and TRPM2 channels. The session was very well attended and the atmosphere vibrant; members of the audience had to resort to standing, reflecting the high level of interest in the speakers’ work and the intriguing nature of these proteins in physiology.

First to speak was Barbara Ehrlich from Yale. Barbara described polycystin‐2 channels as almost exclusively channels of the endoplasmic reticulum membrane. She discussed ideas on the channels as EF hand‐containing calcium‐activated permeation mechanisms which couple to and regulate inositol 1,4,5‐trisphosphate (IP_3_) receptors. She spoke about hypotheses for how mutations in the polycystin‐2 gene might lead to cysts in the kidney, showing interesting 3‐D culture results which suggested that loss of IP_3_ receptor functionality might mediate cyst formation. In the question time there was discussion about how this might arise via changes in polycystin‐2. Her zebrafish and murine studies suggested unexpected roles also in cardiac physiology. Barbara's review with her colleague Yifei Yang nicely summarizes current perspectives on the structural aspects of these channels (Yang & Ehrlich, 2016).

Next was Bertrand Coste from Marseilles. Bertrand described his discovery of Piezo1 and Piezo2 as mechanically activated channels of the plasma membrane and briefly touched on how subsequent studies had shown the importance of Piezo1 in endothelial cell and Piezo2 in Merkel cell mechanical sensing. He explained how the channels apparently have no role in mechanical pain sensation even though the initial discovery was made in sensory neurones. He described his recent extensive mutagenesis work which had revealed a residue involved in ion selectivity and suggested the possibility of a re‐entrant loop in the inner leaflet of the bilayer which might provide the ion pore of the channels (Coste *et al*. 2015).

Felix Viana from Alicante brought us whole‐heartedly onto the topic of pain. He spoke about TRPA1 and gave us important background information, entertainingly handling the controversy over TRPA1's sensitivity to cold. Primarily he focused on his comprehensive and intriguing work on the roles of TRPA1 channels in drug and infection‐induced pain. He showed striking effects of TRPA1 knockout on oxaliplatin‐induced neuropathy in mice and, while acknowledging the seminal work on TLR4 as a receptor for lipopolysaccharide (LPS), he persuasively made the case for rapid actions of LPS on TRPA1 independently of TLR4. New data on lipid A further strengthened the case. His review article provides state of the art analysis of the field (Viana, 2016).

Samantha Pitt from St Andrews took us back to intracellular membranes: this time, the lysosomal membrane and two‐pore channels formed by TPC1 and TPC2. She explained her ideas about how the channels might have distinct functions in the lysosome, suggesting important proton permeability of TPC1 channels. She showed strong pharmacological differences between the channels and raised the possibility that ion selectivity might be modulated by the lipid composition of the lysosomal membrane. She took us through current controversies in the field and proposed a model to bring together apparently contradictory findings; keen discussion followed in the question session. Her review article with colleagues Benedict Reilly‐O'Donnell and Rebecca Sitsapesan puts down in writing several of the key challenges of this hot topic (Pitt *et al*. 2016).

Last but not least, Barbara Miller from Penn‐State brought us back out from the inside of the cell to the plasma membrane, or so we thought. She spoke about her interest in the TRPM2 channel and took us on a journey from what has been classed as a bad channel – a channel of cell death – to a channel which we need and which protects our cells from death. She described how she found deleterious effects of cardiac‐specific knock‐out of TRPM2 in mice subjected to ischaemia–reperfusion, suggesting a protective role of the channels in this stressful context. To explain the findings she developed a compelling case for an intimate relationship between calcium permeability in TRPM2 channels, mitochondrial calcium uptake and reactive oxygen species. Her follow‐up review with Joseph Cheung elegantly presents the case and its wider context (Miller & Cheung, 2016).

So, in the heart of Wales we watched how the ionic dragon fared in the physiologist's den. We wondered at how easily it beguiles and pondered over how we might better understand it. I thank the speakers and audience for making the symposium such a pleasure and I already find myself looking forward to the next time. Whether you could attend or not, I hope you enjoy and learn from the excellent reviews provided by participating speakers (Miller & Cheung, 2016; Pitt *et al*. 2016; Viana, 2016; Yang & Ehrlich, 2016).

## Additional information

### Competing interests

None declared.
